# Optimization of RT-QuIC Assay Duration for Screening Chronic Wasting Disease in White-Tailed Deer

**DOI:** 10.3390/vetsci11020060

**Published:** 2024-02-01

**Authors:** Gokhan Yilmaz, Tamara Morrill, William Pilot, Cian Ward, Gordon Mitchell, Andrei Soutyrine, Hanhong Dan, Min Lin, Jiewen Guan

**Affiliations:** Ottawa Laboratory Fallowfield, Canadian Food Inspection Agency, Ottawa, ON K2J 4S1, Canada

**Keywords:** RT-QuIC, chronic wasting disease, diagnostics, optimization

## Abstract

**Simple Summary:**

Chronic wasting disease (CWD) is a prion disease in cervids that inevitably leads to fatal damage to the nervous system. The spread of CWD has dramatically impacted cervid health and the economic viability of the cervid industry. The real-time quaking-induced conversion (RT-QuIC) assay has shown superior levels of sensitivity in the detection of CWD compared to immuno-based assays. However, methods are rarely explored to determine the optimum RT-QuIC assay duration, a critically important factor affecting the reliability and repeatability of the assay. This study demonstrated and evaluated the use of the receiver operating characteristic (ROC) method to optimize RT-QuIC assay duration using cycle thresholds or max-point ratios to determine assay positivity. Using the optimized assay durations, RT-QuIC produced a significantly higher level of agreement with enzyme-linked immunosorbent assay (ELISA), one of the current diagnostic tools for screening CWD in cervids. Our findings highlighted the significance of optimizing RT-QuIC assay duration for screening CWD.

**Abstract:**

Real-time quaking-induced conversion (RT-QuIC) assays have become a common tool to detect chronic wasting disease (CWD) and are very sensitive provided the assay duration is sufficient. However, a prolonged assay duration may lead to non-specific signal amplification. The wide range of pre-defined assay durations in current RT-QuIC applications presents a need for methods to optimize the RT-QuIC assay. In this study, receiver operating characteristic (ROC) analysis was applied to optimize the assay duration for CWD screening in obex and retropharyngeal lymph node (RLN) tissue specimens. Two different fluorescence thresholds were used: a fixed threshold based on background fluorescence (T_stdev_) and a max-point ratio (maximum/background fluorescence) threshold (T_MPR_) to determine CWD positivity. The optimal assay duration was 33 h for obex and 30 h for RLN based on T_stdev_, and 29 h for obex and 32 h for RLN based on T_MPR_. The optimized assay durations were then evaluated for screening CWD in white-tailed deer from an affected farm. At a replicate level, using the optimized assay durations with T_Stdev_ and T_MPR_, the level of agreement with enzyme-linked immunosorbent assay (ELISA) was significantly higher (*p* < 0.05) than that when using a 40 h assay duration. These findings demonstrate that the optimization of assay duration via a ROC analysis can improve RT-QuIC assays for screening CWD in white-tailed deer.

## 1. Introduction

Chronic wasting disease (CWD) is a transmissible spongiform encephalopathy that affects cervids, such as deer, elk, and moose [1]. As of January 2024, it has been detected in at least 32 states in the United States, four provinces in Canada, Norway, Finland, Sweden, and Republic of Korea [2]. The majority of natural, horizontal CWD transmission occurs through either direct exposure to infectious or misfolded prion proteins (PrP^CWD^) by contact with infected animals or by indirect environmental exposure associated with foraging and rutting [3]. PrP^CWD^ can initiate the conversion of the host’s normal cellular prion protein to its misfolded form, which can then induce amplification of PrP^CWD^ in the local lymphoid tissues, followed by rapid dissemination via the blood or lymphoid cells to systemic lymphoid tissues [3]. Alternatively, PrP^CWD^ may accumulate in peripheral nerves and be transported to the central nervous system without lymphoid amplification. During the disease’s progression, PrP^CWD^ has also been found in many biological tissues, including blood, saliva, urine, skin, muscle, and feces [4,5,6,7]. Thus, PrP^CWD^ is widely used as a diagnostic marker for infected animals. In current CWD diagnostic schemes, enzyme-linked immunosorbent assays (ELISAs) are routinely used as the primary screening assay, followed by confirmation via immunohistochemistry (IHC) [8].

Within the past decade, real-time quaking-induced conversion (RT-QuIC) assays have become common and have shown potential to be used for the routine detection of CWD in cervids [9]. Many RT-QuIC test protocols have been developed and used for the detection of PrP^CWD^ in a range of biological tissues and environmental samples [5,6,7,10,11,12]. The RT-QuIC assay exploits the ability of infectious or misfolded prion proteins to seed the conversion of monomeric prion protein substrates into the misfolded isoform. These misfolded monomers can then form larger amyloid fibrils, which are then detected by the amyloid-sensitive fluorescent dye thioflavin T (ThT) [13]. As such, the RT-QuIC assay is extremely sensitive and able to detect sub-femtograms of misfolded prion proteins provided the time (or cycles) for target amplification is sufficient [14,15]. However, the monomeric prion protein substrates tend to self-aggregate and may produce false positive ThT signals when the assay time is prolonged or the number of cycles increases [16,17]. A wide range of assay times from 24 to 62.5 h have been used in various RT-QuIC protocols for detecting CWD in different sample matrices [5,6,7,10,11,12], but information on how to determine the appropriate assay length is very limited. To reduce false-positive outcomes while maintaining sensitivity, methods are needed for the optimization of the assay duration of specific RT-QuIC assays for screening or diagnostic applications.

In the above RT-QuIC protocols, to determine CWD positivity, a fluorescent threshold (T_stdev_) was defined as a few (e.g., 5/10 [5,6,7,8,11,18]) standard deviations above the average background fluorescence of all reactions. Specimens have been classified as CWD-positive using a probability test, in which a specimen was positive when a certain number (e.g., ≥4 out of 8 [5,8]) of replicates surpassed T_stdev_. Additionally, the Mann–Whitney U-test has been used to determine CWD positivity by comparing the cycle thresholds (Ct) or rates (the reciprocals of cycle thresholds) of replicate reactions of a specimen with those of the negative controls [6]. Recently, the use of max-point ratios (the maximum fluorescence/background fluorescence, MPR) has been proposed to improve the consistency of RT-QuIC analyses [9,12]. With the MPR, specimens were classified as CWD-positive using Welch’s analysis of variance (ANOVA) by comparing the MPR values of unknown specimens against those of a known negative control. In addition, an MPR-based threshold (T_MPR_), which may better account for initial variations between wells, was proposed as an alternative to an independent T_stdev_ per reaction plate. Building on the above applications of T_stdev_ or T_MPR_ in the determination of CWD positivity, this study proposed and demonstrated the application of a receiver operating characteristic (ROC) analysis [19] for optimizing the RT-QuIC assay duration for CWD screening. ROC analyses have been widely applied for the evaluation of tests with dichotomous outcomes (positive/negative test results), using sensitivity and specificity as measures of accuracy in comparison with the gold standard [19]. A ROC curve is a plot of sensitivity versus the false-positive rate at varying thresholds, and the area under the curve (AUC) is an effective measure of classifying power [19]. In this study, RT-QuIC data from control tissue specimens (obex and retropharyngeal lymph nodes (RLN) as examples) with known CWD statuses confirmed by IHC were used to construct ROC curves to optimize the assay duration under specific conditions in our laboratory. The optimized assay durations were further evaluated for the screening of PrP^CWD^ in white-tailed deer from a CWD-affected farm against ELISA, a widely used screening tool.

## 2. Materials and Methods

### 2.1. Sample Preparation

**Figure 1:**
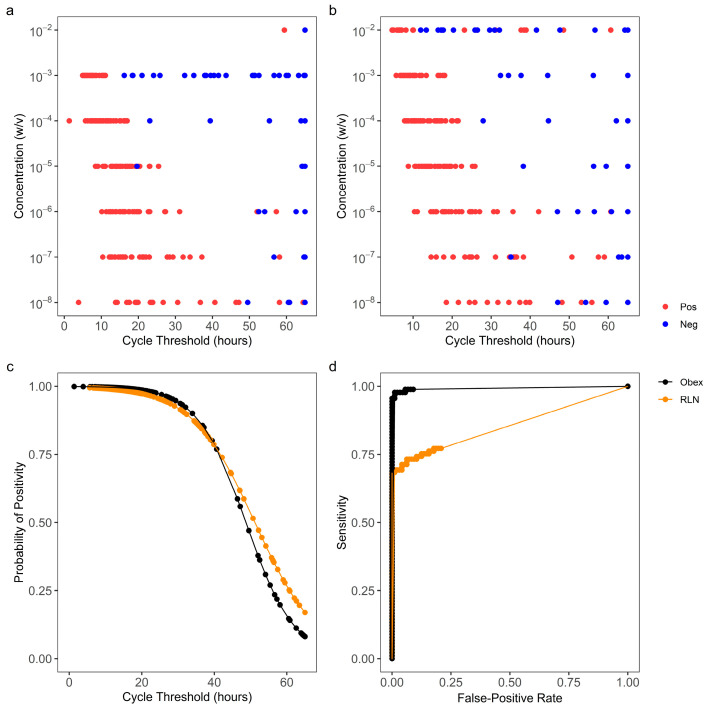
Receiver operating characteristic curve (ROC) analysis to optimize real-time quaking-induced conversion (RT-QuIC) assay duration to detect chronic wasting disease (CWD) with T_stdev_. Cycle thresholds (Ct) were calculated for RT-QuIC reactions seeded by gradient concentrations of CWD+ and CWD− obex (**a**) and retropharyngeal lymph node (RLN) (**b**) tissue specimens. Cycle threshold was the time when the thioflavin T (ThT) signal of a reaction surpassed T_stdev_, which was defined as the average baseline (the 1st cycle) reading of all the reactions in relative fluorescence units (RFU) plus 10 standard deviations. A cycle threshold of 65 h was assigned to a reaction from which no ThT signal surpassed T_stdev_. Based on the cycle thresholds for obex (**a**) and RLN (**b**) and the known CWD status of the reactions, classification prediction models (**c**) and the corresponding ROC curves (**d**) were constructed, using the 10^−4^ to 10^−8^ *w*/*v* dilutions for obex and the 10^−3^ to 10^−8^ *w*/*v* dilutions for RLN.

Obex and RLN tissue specimens from white-tailed deer testing positive or negative for CWD (CWD+ or CWD−) using IHC as described in [20] were used as controls in this study for the determination of optimal assay durations. These tissue specimens were homogenized in grinding tubes containing quarter-inch grinding beads using a Precellys 24 Tissue Homogenizer (Bertin, Paris, France) to form 10% or 15% *w*/*v* homogenates in 0.05% sodium dodecyl sulfate (SDS). The homogenates were serially diluted 10-fold in 0.05% SDS to generate a gradient concentration ranging from 1.0 × 10^−2^ to 1.0 × 10^−11^ *w*/*v*. To generate the cycle threshold and MPR distributions (Figure 1, Figures S1a–c and S2a–c), there were between 6 and 42 replicates. The number of replicates per condition are shown in Table S1. Obex and RLN tissue specimens collected from white-tailed deer with unknown CWD status were homogenized the same way as described above and diluted in 0.05% SDS to form 1.0 × 10^−4^ *w*/*v* homogenates for RT-QuIC and ELISA tests as described below.

### 2.2. Production of Recombinant Prion Protein

Recombinant Syrian hamster prion protein (PrP^rec^), with amino acids 90–231, was prepared as described in [21]. In brief, protein expression in *Escherichia coli* Rosetta^TM^ (DE3) culture was induced using the Overnight Express Autoinduction System 1—Novagen kit (EMD Millipore, Darmstadt, Germany). Inclusion bodies were harvested using the BugBuster^®^ Master Mix (EMD Millipore) following the manufacturer’s protocol. The inclusion bodies were solubilized in denaturation buffer (8 M guanidine hydrochloride, 0.1 M sodium phosphate monobasic, and 0.01 M Tris pH 8.0) for 1 h at room temperature. The solubilized protein was bound to Ni-NTA Superflow resin (Qiagen, Venlo, the Netherlands) and refolded with a linear gradient from 100% denaturation buffer to 100% refolding buffer (0.1 M sodium phosphate monobasic and 0.01 M Tris pH 8.0) flowing at 1.5 mL min^−1^ over 3 h. The protein was eluted with a linear gradient from 100% refolding buffer to 100% elution buffer (0.5 M imidazole, 0.1 M sodium phosphate monobasic, and 0.01 M Tris pH 5.6) at 2.0 mL min^−1^ over 40 min. The eluted protein was dialyzed (0.05 M sodium phosphate monobasic/dibasic buffer pH 7.3) overnight and the following day twice over 2 h periods in fresh dialysis buffer. PrP^rec^ was stored at −80 °C before its use.

### 2.3. RT-QuIC Methods

RT-QuIC assays were performed as previously described [22], with slight modifications. Briefly, 95 µL of the reaction master mix and 5 µL of diluted obex or RLN tissue homogenate were added to each well of a 96-well plate. Wells contained 300 mM NaCl, 1 mM EDTA, 10 µM ThT, 0.1 mg/mL PrP^rec^, and 50 mM Na_3_PO4 (pH 7.2–7.4). The reactions were run using BMG FluoStar^®^ plate readers (BMG Labtech, Ortenberg, Germany). Assays were performed at 42 °C for 65 h for the determination of optimal assay duration or using the optimized assay durations. Each cycle lasted approximately 17 min, with 7 repeats of a 1 min shake at 700 rpm (double orbital) and a 1 min rest, followed by a 1 min reading. ThT fluorescence measurements were taken every cycle at a gain of 1200, excitation of 450 nm, and emission of 480 nm. The assay data was exported from the Mars data analysis software (BMG Labtech) and processed in Microsoft Excel (Microsoft 365) and RStudio (version 2023.06.02+534).

### 2.4. Determination of Optimal Assay Duration for T_stdev_

The approach described by Gray et al. [16] was followed to determine the optimal assay duration. In brief, distributions of RT-QuIC cycle thresholds were generated using reactions that were seeded with 5 control CWD+ and 5 control CWD− obex and RLN tissue homogenates in 10-fold serial dilutions. The cycle threshold was defined as the time when the ThT signal of a reaction surpassed T_stdev_, which was calculated using the average baseline or first cycle reading of all the reactions in relative fluorescent units (RFU) plus 10 standard deviations. A cycle threshold of 65 h was assigned for reactions in which the ThT signal did not cross the threshold within the 65 h assay. Cycle threshold was used as a binary classifier for CWD positivity, and ROC curve analyses were conducted comparing cycle threshold against the known CWD status of the tissue homogenates. The optimal assay durations were defined as the threshold with the highest Youden index (sensitivity + specificity–1) [23]. In the case of more than one assay duration with the highest Youden index, the mean of the assay durations was considered optimal. ROC curve calculations were performed with the RStudio ROCR package (version 1.0-11) [24].

### 2.5. Determination of Optimal Assay Duration Time for T_MPR_

Determination of optimal assay duration time for T_MPR_ was based on the same ThT data from the above RT-QuIC reactions that were seeded with the control CWD+ and CWD− brain and RLN tissue homogenates. MPR was defined as the ratio of maximum RFU to background (4th cycle) RFU by Rowden et al. [9]. In this study, MPR was calculated for each cycle using the maximum accumulated RFU, from the 4th cycle (52 min of the assay) to the 224th cycle (65 h), using the maximum accumulated RFU within the corresponding cycles, and thus, 221 MPRs were calculated from each reaction. For each cycle, a ROC curve using the accumulated MPR as a binary classifier for CWD was constructed against the known CWD status of tissue homogenates. Therefore, 221 ROC curves were generated. The area under the ROC curve (AUC) was plotted against the assay duration of the corresponding cumulative assay cycles. As the AUC plateaued past ~30 h (Figure S3), the optimal assay duration was chosen as the point at which dAUC/dT stopped increasing (i.e., consistently less than 0.01). The T_MPR_ for each ROC curve was then determined by finding the Youden index for that particular ROC curve. The T_MPR_ was defined as the highest T_MPR_ in the accumulated cycles.

### 2.6. Evaluation of the Optimized Assay Durations

The optimized assay durations were evaluated with obex and RLN tissue specimens that were collected from 104 white-tailed deer in a Canadian cervid farm affected by CWD. The tissue specimens were homogenized the same way as described above to form 1.0 × 10^−4^ *w*/*v* homogenates for RT-QuIC and 20% obex and 15% RLN homogenates for ELISA. ELISA tests were performed following the procedure in [25], using the TeSeE Purification kit and the TeSeE SAP Detection kit (Bio-rad, Hercules, CA, USA). RT-QuIC tests were performed as previously described using the optimal assay durations. Obex and RLN tissue specimens that were ELISA-positive or had any sample replicates that tested positive for CWD by RT-QuIC were tested by IHC for confirmation.

The classification of specimens tested by RT-QuIC was carried out using the Mann–Whitney U-test and the probability test using T_stdev_ [16], as well as the Welch’s *t*-test and the probability test using T_MPR_ [9]. Cycle threshold or MPR from quadruplicate RT-QuIC reactions on each tissue specimens were compared with the negative controls with the Mann–Whitney U-test and Welch’s *t*-test using R. For the probability test, tissue specimens were classified as negative if no replicates surpassed the threshold, positive if all 4 replicates surpassed the threshold, and suspect if at least 1 out of 4 replicates surpassed the threshold. Suspect specimens were re-tested in quadruplicate and then classified positive if at least 4 out of 8 replicates surpassed the threshold. Kappa analysis was used to test agreement between RT-QuIC and ELISA in R following the method in [26].

To further evaluate the optimal assay durations, the quadruplicate RT-QuIC reactions performed for the 104 obex and RLN tissue specimens were extended to a 40 h assay duration, which was used for detection of CWD in white-tailed deer obex and lymph nodes [8] and compared to their ELISA results. The ideal assay durations and 40 h assay duration were further compared at a replicate level using a McNemar’s test, using the ideal assay duration classification as the expected results and the 40 h assay duration classification as the observed results.

## 3. Results

### 3.1. Optimization of RT-QuIC Assay Durations Based on T_stdev_

Obex and RLN tissue specimens with known CWD statuses were serially diluted to simulate specimens containing various concentrations of PrP^CWD^. At 10^−2^ *w*/*v*, the obex tissue homogenate inhibited the RT-QuIC reactions and prevented the production of cycle thresholds based on the T_stdev_ within the 65 h assay (Figure 1a). For RLN, at 10^−2^ *w*/*v*, non-specific amyloid formation occurred as early as 12 h (Figure 1b). From 10^−4^ to 10^−8^ *w*/*v* for obex and 10^−3^ to 10^−8^ *w*/*v* for RLN, the cycle thresholds for the CWD+ tissue homogenates occurred earlier than those for the CWD− homogenates (Figure 1a,b). At 10^−9^ *w*/*v* and lower, the cycle thresholds of most reactions seeded with either the CWD+ or CWD− homogenates were close to 65 h, suggesting an analytical sensitivity or limit of detection of CWD in obex and RLN tissue specimens at 10^−8^ for both tissues. Thus, a global ROC curve, including dilutions from 10^−4^ to 10^−8^ *w*/*v* and from 10^−3^ to 10^−8^ *w*/*v*, was constructed for obex and RLN, respectively (Figure 1c,d). The observed AUCs were 0.933 and 0.890 for obex and RLN, respectively (Figure 1d), indicating that cycle threshold has a very strong classifying power for both types of tissues. Based on these ROC curves, the optimal assay duration time was 33 h for obex and 30 h for RLN, respectively.

### 3.2. Optimization of RT-QuIC Assay Durations Based on T_MPR_

**Figure 2:**
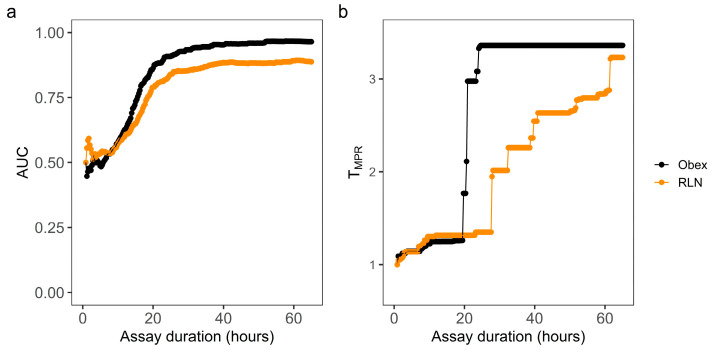
ROC analysis of T_MPR_ as a classifier using reactions seeded by gradient concentrations of of CWD+ and CWD− obex and RLN tissue specimens at varying assay durations. Max-point ratio (MPR) was defined as the ratio of RFU within the assay duration to the background (the 4th cycle) RFU. Individual ROC curves were constructed for each of 221 assay durations corresponding to each addition of one cycle from the 4th to 224th cycle. The area under ROC curve (AUC) (**a**) and the MPR threshold (T_MPR_) (**b**) based on each of the 221 ROC curves were plotted against the corresponding assay duration.

The same ThT data from the above RT-QuIC reactions were used to calculate MPR values for each reaction along with the increasing assay durations. The MPR values for the CWD+ obex or RLN tissue homogenates at 10^−2^ *w/v* were indistinguishable from those for the CWD− tissue homogenates at various assay durations (Figures S1a–c and S2a–c), reflecting the inhibited or non-specific amyloid formations as described above. Thus, the MPR values at this concentration were not used for the determination of the optimal assay duration. For obex from 10^−4^ to 10^−8^ *w*/*v* and RLN from 10^−3^ to 10^−8^ *w*/*v*, the MPR values from the reactions seeded with the CWD+ homogenates became greater than those with the CWD− homogenates at assay durations of 28 and 46 h (Figures S1b,c and S2b,c). As such, RFU data generated from these concentrations were used to construct the MPR distributions and global ROC curves. Based on the MPR distribution corresponding to each increasing assay cycle from the 4th to 224th cycle, 221 ROC curves were constructed for the obex or RLN tissue homogenates. These ROC curves indicated that MPR has very strong classifying power when the assay duration is 28 h or longer. Such findings were confirmed by plotting the AUC values of the 221 ROC curves against the corresponding assay durations for obex or RLN (Figure 2a). However, the classifying power of MPR plateaus after around 30 h. Thus, the optimal assay durations were defined as the point at which the dAUC/dT stopped increasing (Figure S3). As such, an assay duration of 102 cycles (29 h) for obex and 110 cycles (32 h) for RLN were considered optimal for the detection of CWD with MPR. Based on the individual ROC curves, an accumulated MPR threshold (T_MPR_) was determined and plotted against assay duration (Figure 2b). The T_MPR_ corresponding to the optimal assay duration was 3.36 and 2.00 for obex and RLN, respectively. At the pre-determined assay duration of 40 h, the ideal T_MPR_ was 3.36 and 2.54 for obex and RLN, respectively.

### 3.3. Evaluation of the Optimized RT-QuIC Assay Durations

To evaluate the optimized RT-QuIC assay durations, obex and RLN tissue specimens from 104 white-tailed deer in a CWD-affected farm were tested by RT-QuIC and ELISA. The specimens that were classified as RT-QuIC-positive by the Mann–Whitney test or Welch’s *t*-test or those that had at least one replicate that surpassed T_stdev_ or T_MPR_ are listed in Tables S2 and S3. Suspect samples with any replicates that surpassed T_stdev_ or T_MPR_ were further tested by IHC for confirmation, and only the ELISA-positive specimens were positive by IHC. When using cycle threshold as the classifier for RT-QuIC, classifications with the Mann–Whitney U test was in 100% agreement with the ELISA results for both the obex and RLN tissue specimens (Table 1). When using MPR as the classifier for RT-QuIC, CWD classification using Welch’s *t*-test was 98.1% (κ = 0.823, 95% CI: 0.581–1) and 92.3% (κ = 0.558, 95% CI: 0.242–0.874) in agreement with ELISA for the obex and RLN tissue specimens, respectively (Table 1 and Table S2). The classification using the probability test based on T_stdev_ and T_MPR_ was in 100% agreement with ELISA for both the obex and RLN tissue specimens (Table 1 and Table S2).

**Figure 3:**
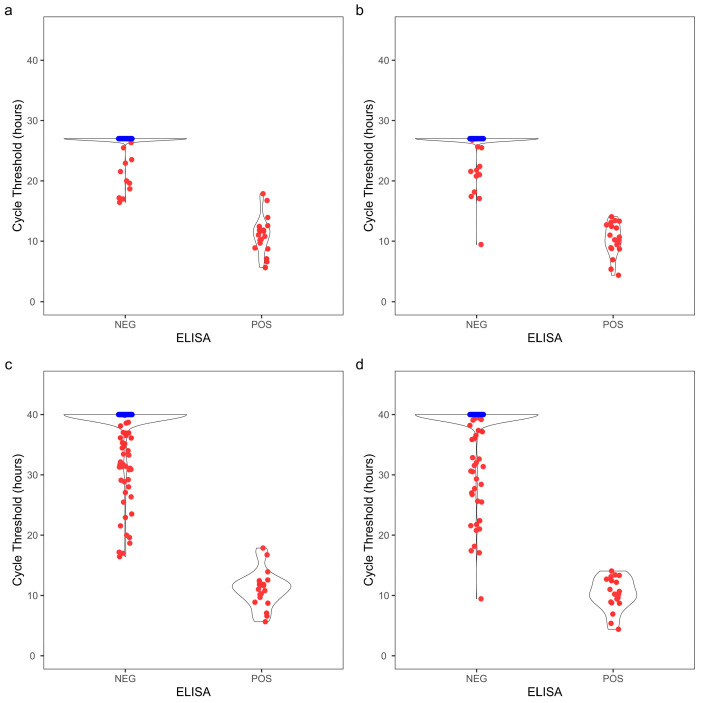
Cycle threshold distributions of 104 obex and RLN white-tailed deer samples from a CWD-affected farm. Ct values from quadruplicate reactions from 104 samples were determined at 33 h with obex (**a**), 30 h with RLN (**b**), 40 h with obex (**c**), and 40 h with RLN (**d**). Replicates that did not surpass T_stdev_ within the assay duration were assigned a Ct of 33 h (**a**), 30 h (**b**), or 40 h (**c**,**d**). Replicates in red surpassed T_stdev_ within the assay duration, whereas replicates in blue did not surpass T_stdev_.

**Figure 4:**
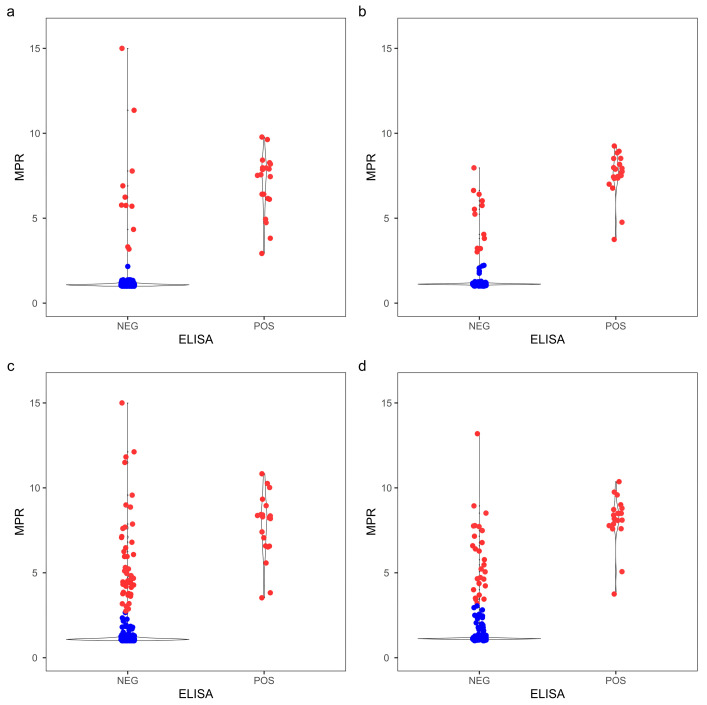
Max-point ratio distributions of 104 obex and RLN white-tailed deer samples from a CWD-affected farm. MPR values from quadruplicate reactions from 104 samples were determined at 102 cycles (29 h) with obex (**a**), 110 cycles (32 h) with RLN (**b**), 40 h with obex (**c**), and 40 h with RLN (**d**). Replicates in red surpassed T_MPR_ within the assay duration, whereas replicates in blue did not cross T_MPR_. T_MPR_ was defined as 3.36 and 2.00 for obex and RLN at optimal durations, respectively, and 3.36 and 2.54 at 40 h for obex and RLN, respectively.

To further evaluate the optimized assay durations, the RT-QuIC assay was extended to 40 h for comparison. The distribution of cycle threshold and MPR for both obex and RLN clearly showed that more ELISA-negative replicates became CWD+ by RT-QuIC when the assay duration was prolonged to 40 h compared to the optimized duration (Figure 3 and Figure 4). The agreement between RT-QuIC and ELISA dropped by about 5% for all the conditions when increasing the assay duration to 40 h (Table S4). Contingency tables were constructed at a replicate level comparing the optimized assay durations with the 40 h assay duration (Table S5), and the RT-QuIC results were significantly different with both T_Stdev_ (*p*_obex_ = 2.54 × 10^−8^, *p*_RLN_ = 3.64 × 10^−5^) and T_MPR_ (*p*_obex_ = 2.00 × 10^−7^, *p*_RLN_ = 2.57 × 10^−3^) using McNemar’s test.

## 4. Discussion

The RT-QuIC assay is extremely sensitive and able to detect sub-femtograms of infectious or misfolded prion proteins, which seed the conversion or aggregation of monomeric prion substrates to form larger amyloid fibrils [14,15]. The kinetics of amyloid fibril formation are affected by the types and concentrations of salts [27] or solvents [28] in the assay solutions and also by the assay temperatures and shaking arrangements [28]. As such, a wide range of assay durations have been used in RT-QuIC for the detection of CWD [5,6,7,10,11,12]. In general, a longer assay duration results in a higher level of sensitivity but a lower level of specificity due to the self-aggregation of the prion protein substrates [16,17]. To obtain the best combination of sensitivity and specificity, this study proposed and demonstrated the use of the ROC analysis for optimizing the RT-QuIC assay duration to screen CWD in cervids.

The optimization of the RT-QuIC assay duration was performed for both T_stdev_ and T_MPR_, two thresholds that are currently used in the classification of CWD. Considering that specimens contain various concentrations of infectious prion protein, pooled ThT signals generated from serial dilutions of the control specimens were used in the ROC analysis for determining the optimal assay duration, instead of identifying the individual cut-off values for the assay durationat various dilutions as described in [16]. To demonstrate this analysis, our study used serial dilutions from five CWD-positive and five CWD-negative specimens that were confirmed by IHC as controls to construct the ROC curves. Five samples were found to be necessary assuming type I error (α < 0.05) and type II error (β < 0.05), an AUC of 0.975, and a null hypothesis AUC of 0.5 with the ratio of the sample sizes in the negative/positive groups being equal to 1 [29]. Field samples would be the ideal option for ROC curve construction, but a much larger sample size with a wide distribution of PrP^CWD^ levels would be required to achieve the same level of type I and II errors. Moreover, the required sample size would depend on the prevalence of CWD in the specific area, which can reach as high as 15.8% in Alberta, Canada [30]. The results by RT-QuIC using the optimized assay duration based on T_stdev_ were in 100% agreement with those attained by the widely used ELISA for screening CWD in obex and RLN tissue specimens collected from the affected white-tailed deer farm. In comparison, more replicates from specimens that were tested as CWD− ELISA became CWD+ by RT-QuIC when the assay duration was extended to 40 h, which was used for the detection of CWD in white-tailed deer obex and lymph nodes [8]. Based on the IHC confirmation, RT-QuIC using the optimized assay durations and thresholds produced significantly fewer false-positive replicates compared to the number of replicates using 40 h and thus would limit the costs and labor associated with retesting the samples. It is possible that RT-QuIC might detect minute amounts of infectious prion protein that were missed by IHC, and a ROC analysis based on mouse bioassay data could be an option for further inquiry [16]. Nonetheless, our findings demonstrated the effectiveness of using ROC and AUC analyses for optimizing RT-QuIC assay durations in screening CWD as an alternative to ELISA. As many factors may affect RT-QuIC performance [9,27,28], it is expected that the optimal assay duration may vary for different sample matrixes, with different substrates and reagents, and using different fluorometers. In addition, with ROC curves, there are multiple methods to interpret the optimal threshold cut-offs, such as the Youden index and the point closest to the (0,1) method [31]. These different methods can lead to slightly different “optimal” cut-offs. If needed, the threshold cut-offs can be weighted towards sensitivity or specificity, such as in the case of a screening assay or a confirmatory assay, respectively. Nonetheless, tools like ROC analysis can be helpful in enhancing RT-QuIC for CWD detection.

In CWD classification the Mann–Whitney U-test and the probability test have been used based on T_stdev_, and Welch’s *t*-test and the probability test based on T_MPR_ [9,16]. In this study, based on T_stdev_, the Mann–Whitney U-test and the probability test produced consistent results for screening CWD in the obex and RLN specimens from the affected farm. However, based on T_MPR_, the probability test performed better than that of Welch’s *t*-test for screening both the obex and RLN specimens. Self-aggregation of the protein substrates may have occurred at low levels in the false-positive reactions. Although the ThT signals from these self-aggregations were not high enough to produce MPRs that surpassed T_MPR_, they were significantly higher than the signals from the negative controls. Our findings suggested that the application of the T_MPR_ and the probability test might help prevent false-positive results derived from the low levels of self-aggregation of the prion substrates.

Overall, this study proposed and demonstrated the use of a ROC analysis to optimize RT-QuIC assay duration based on both T_stdev_ and T_MPR_. With T_stdev_, the optimal assay durations were 33 h for obex and 30 h for RLN. With T_MPR_, the optimal assay durations were 29 h for obex and 32 h with RLN, respectively. The optimized assay durations were evaluated and proven to be effective in RT-QuIC applications for screening CWD in obex and RLN tissue specimens of white-tailed deer when compared to ELISA, the widely used screening assay. The findings suggest the potential of optimizing RT-QuIC assay duration for enhancing CWD detection in various animal specimens and environmental samples.

## Figures and Tables

**Table 1:** Screening chronic wasting disease (CWD) in obex and retropharyngeal lymph node (RLN) tissue specimens in 104 white-tailed deer by RT-QuIC and ELISA.

			With T_Stdev_				With T_MPR_			
			Mann–Whitney ^1^		Probability Approach ^2^		Welch *t*-Test ^3^		Probability Approach ^4^	
			Pos.	Neg.	Pos.	Neg.	Pos.	Neg.	Pos.	Neg.
Obex	ELISA	Pos.	5	0	5	0	5	0	5	0
		Neg.	0	99	0	99	2	97	0	99
RLN	ELISA	Pos.	5	0	5	0	5	0	5	0
		Neg.	0	99	0	99	7	92	0	99

^1.^ Obex and RLN specimens were homogenized, diluted to 10^−4^ (*w*/*v*), and then tested RT-QuIC using 33 h and 30 h assay durations, respectively. The cycle thresholds of quadruplicate reactions from each specimen were compared with those from a negative control specimen using a Mann–Whitney U-test, and a specimen was classified as positive when *p* < 0.05. Cycle threshold was the time when the ThT signal of a reaction surpassed T_stdev_, which was the average baseline (the 1st cycle) reading of all the reactions in RFU plus 10 standard deviations. ^2.^ Tissue specimens were classified as negative if none of the 4 replicates surpassed T_stdev_, positive if all 4 replicates surpassed T_stdev_, and suspect if at least 1 out of 4 replicates surpassed the threshold. Suspect specimens were re-tested in quadruplicate and then classified positive if at least 4 out of 8 replicates surpassed T_stdev_. ^3.^ The MPRs of quadruplicate reactions from each specimen were compared with those from a negative control specimen using Welch’s *t*-test, and a specimen was classified as positive when *p* < 0.05. MPR was defined as the ratio of maximum RFU to background (the 4th cycle) RFU within the 29 h and 32 h assay durations for obex and RLN specimens, respectively. ^4.^ Tissue specimens were classified as negative if none of the 4 replicates surpassed T_MPR_, positive if all 4 replicates surpassed T_MPR_, and suspect if at least 1 out of 4 replicates surpassed T_MPR_. Suspect specimens were re-tested in quadruplicate, and then classified positive if at least 4 out of 8 replicates surpassed T_MPR_. T_MPR_ was 3.36 and 2.00 for obex and RLN tissue homogenates, respectively.

## Data Availability

The original contributions presented in the study are included in the article and the Supplementary Material. Further inquiries can be directed to the corresponding author. The RStudio scripts to conduct the study can be accessed at github.com/gyilm039/Optimizing-RT-QuIC-Sample-Classification.

## Supplementary Materials

The following supporting information can be downloaded at: https://www.mdpi.com/article/10.3390/vetsci11020060/s1, Figure S1: Examples of distributions of real-time quaking-induced conversion (RT-QuIC) max-point ratios (MPR) with obex homogenates at various assay durations; Figure S2: Distributions of real-time quaking-induced conversion (RT-QuIC) max-point ratios (MPR) with retropharyngeal lymph node homogenates (RLN) at various assay durations; Figure S3: Rate of change of area under the curve (AUC) of receiver operating characteristic (ROC) curves constructed with max-point ratios (MPR) with increasing assay durations; Table S1: Number of obex and retropharyngeal lymph node (RLN) replicates used to construct receiver operating characteristic (ROC) curves to optimize real-time quaking-induced conversion (RT-QuIC) assay duration for detecting chronic wasting disease (CWD); Table S2: Screening chronic wasting disease (CWD) in 104 white-tailed deer by RT-QuIC based on cycle threshold and enzyme-linked immunosorbent assay (ELISA); Table S3: Screening chronic wasting disease in 104 white-tailed deer by RT-QuIC based on MPR and ELISA; Table S4: Replicate-level comparison of RT-QuIC screening of 104 white-tailed deer at various assay durations and classifier thresholds; Table S5: Contingency tables for a replicate-level McNemar test between optimized assay durations and a 40 h assay duration.
