# Supplementary material for: Optimization of RT-QuIC Assay Duration for Screening Chronic Wasting Disease in White-Tailed Deer
Source: Vet Sci. 2024 Feb 1;11(2):60. doi: 10.3390/vetsci11020060 (PMC10891863; doi:10.3390/vetsci11020060)
Supplement: Supplementary file 1 [file vetsci-11-00060-s001.zip › Supplementary Figure 1.pdf]

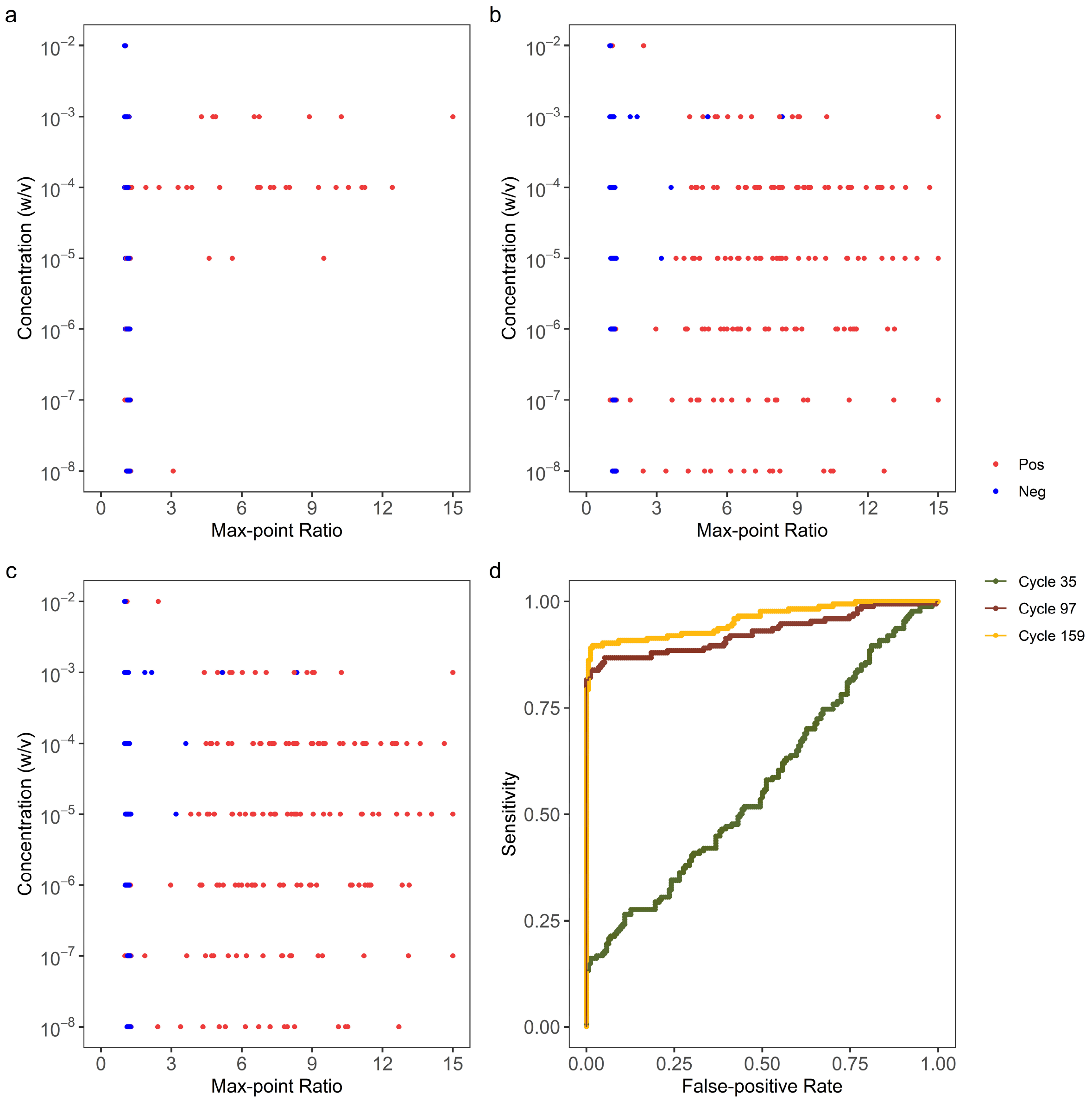

Supplementary Figure 1: Examples of distributions of real-time quaking-induced conversion (RT-QuIC) max-point ratios (MPR) with obex homogenates at various assay durations. MPR distributions of CWD- and CWD+ controls of a gradient concentration were generated for assay durations corresponding to the a) 35th (10 hours), b) 97th (28 hours), and c) 159th (46 hours) cycle and were capped at 15 for a)-c). Receiver operating characteristic curves (d) were constructed using replicates from the 10^{-4} to 10^{-8} w/v concentrations to show the classifying power of MPR with obex at various assay durations.
