# Supplementary material for: Optimization of RT-QuIC Assay Duration for Screening Chronic Wasting Disease in White-Tailed Deer
Source: Vet Sci. 2024 Feb 1;11(2):60. doi: 10.3390/vetsci11020060 (PMC10891863; doi:10.3390/vetsci11020060)
Supplement: Supplementary file 1 [file vetsci-11-00060-s001.zip › Supplementary Figure 3.pdf]

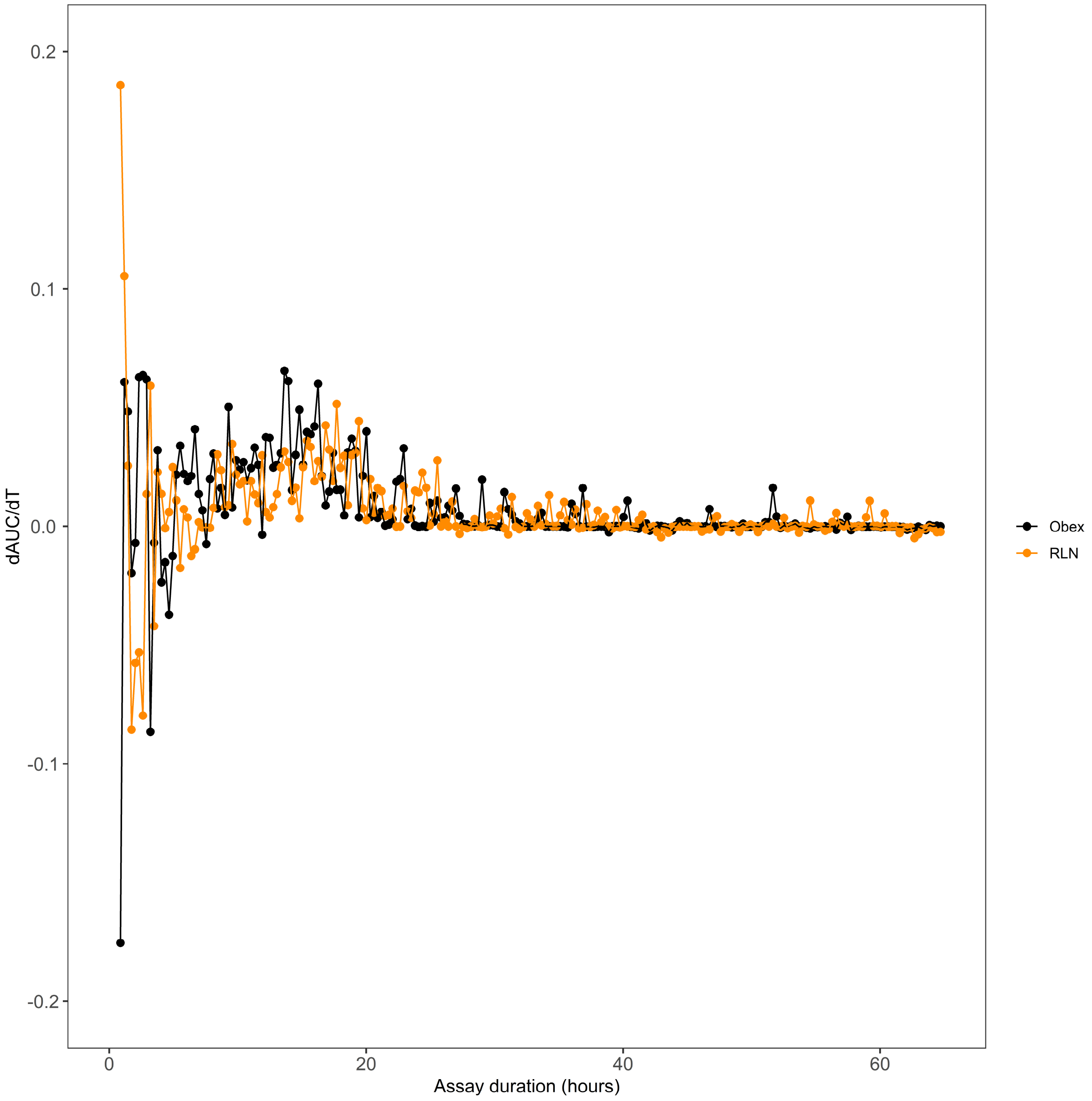

Supplementary Figure 3: Rate of change of area under the curve (AUC) of receiver operating characteristic (ROC) curves constructed with max-point ratio (MPR) with increasing assay durations. ROC curves were constructed using MPR distributions of CWD- and CWD+ controls of a gradient concentration (10^{-4} to 10^{-8} for obex, 10^{-3} to 10^{-8} for RLN) with increasing duration. The rate of change of AUC with each addition of a real-time quaking-induced conversion cycle is shown against the assay duration.
