# Supplementary material for: Optimization of RT-QuIC Assay Duration for Screening Chronic Wasting Disease in White-Tailed Deer
Source: Vet Sci. 2024 Feb 1;11(2):60. doi: 10.3390/vetsci11020060 (PMC10891863; doi:10.3390/vetsci11020060)
Supplement: Supplementary file 1 [file vetsci-11-00060-s001.zip › Supplementary Tables.pdf]

Table S1: Number of obex and retropharyngeal lymph node (RLN) replicates used to construct receiver operating characteristic (ROC) curves to optimize real-time quaking-induced conversion (RT-QuIC) assay duration for detecting chronic wasting disease (CWD)

Concentration (w/v)	Obex Replicates	RLN Replicates
10^{-2}	30	42
10^{-3}	36	42
10^{-4}	42	42
10^{-5}	36	42
10^{-6}	36	42
10^{-7}	30	42
10^{-8}	30	42
10^{-9}	30	30
10^{-10}	6	0
10^{-11}	6	0

Five CWD- and five CWD- obex and retropharyngeal node specimens that were confirmed by immunohistochemistry were used to generate distributions of RT-QuIC cycle thresholds and max-point ratios. The number of replicates denotes the number of technical replicates per tissue at a specific concentration for both positive and negative specimens. To construct the ROC curves, the 10^{-4} to 10^{-8} dilutions were used for obex, while the 10^{-3} to 10^{-8} dilutions were used for RLN, and at each dilution at least 6 replicates from each specimen were included.

Table S2: Screening chronic wasting disease (CWD) in 104 white-tailed deer by RT-QuIC based on cycle threshold[1] and enzyme-linked immunosorbent assay (ELISA)

	RT-QuIC				
	Mann-Whitney U Test		Probability test		
ID[2]	P-value	Pos/Neg	#Pos/#tested[3]	Pos/Neg	ELISA
Obex-1	0.227	Neg	1/8	Neg	Neg
Obex-7	0.011	Pos	4/4	Pos	Pos
Obex-8	0.227	Neg	3/8	Neg	Neg
Obex-18	0.227	Neg	1/8	Neg	Neg
Obex-19	0.011	Pos	4/4	Pos	Pos
Obex-20	0.011	Pos	4/4	Pos	Pos
Obex-25	0.227	Neg	1/8	Neg	Neg
Obex-26	0.227	Neg	1/8	Neg	Neg
Obex-28	0.227	Neg	1/8	Neg	Neg
Obex-29	0.093	Neg	2/8	Neg	Neg
Obex-34	0.227	Neg	2/8	Neg	Neg
Obex-35	0.227	Neg	1/8	Neg	Neg
Obex-37	0.227	Neg	1/8	Neg	Neg
Obex-39	0.227	Neg	1/8	Neg	Neg
Obex-45	0.011	Pos	4/4	Pos	Pos
Obex-46	0.227	Neg	1/8	Neg	Neg
Obex-55	0.011	Pos	4/4	Pos	Pos
Obex-56	0.227	Neg	2/8	Neg	Neg
Obex-68	0.876	Neg	1/8	Neg	Neg
Obex-71	0.876	Neg	1/8	Neg	Neg
Obex-73	0.876	Neg	1/8	Neg	Neg
Obex-75	0.690	Neg	2/8	Neg	Neg
Obex-78	0.876	Neg	1/8	Neg	Neg
Obex-79	0.876	Neg	1/8	Neg	Neg
Obex-80	0.796	Neg	2/8	Neg	Neg
Obex-82	0.779	Neg	2/8	Neg	Neg
Obex-84	0.876	Neg	1/8	Neg	Neg
Obex-97	0.500	Neg	3/8	Neg	Neg
Obex-98	0.647	Neg	1/8	Neg	Neg
Obex-100	0.500	Neg	1/8	Neg	Neg
RLN-7	0.013	Pos	4/4	Pos	Pos
RLN-8	0.062	Neg	3/8	Neg	Neg
RLN-9	0.500	Neg	1/8	Neg	Neg
RLN-18	0.647	Neg	1/8	Neg	Neg
RLN-19	0.013	Pos	4/4	Pos	Pos
RLN-20	0.013	Pos	4/4	Pos	Pos
RLN-21	0.500	Neg	1/8	Neg	Neg
RLN-28	0.500	Neg	1/8	Neg	Neg

RLN-38	0.500	Neg	1/8	Neg	Neg
RLN-39	0.500	Neg	1/8	Neg	Neg
RLN-40	0.500	Neg	1/8	Neg	Neg
RLN-45	0.011	Pos	4/4	Pos	Pos
RLN-55	0.011	Pos	4/4	Pos	Pos
RLN-61	0.093	Neg	2/8	Neg	Neg
RLN-77	0.227	Neg	1/8	Neg	Neg
RLN-88	0.227	Neg	1/8	Neg	Neg
RLN-92	0.227	Neg	1/8	Neg	Neg
RLN-100	0.227	Neg	1/8	Neg	Neg

1. Obex and retropharyngeal lymph node tissue specimens from 104 white-tailed deer were tested by RT-QuIC in quadruplicate with an assay duration of 33 hours and 30 hours, respectively. CWD positivity was determined using the Mann-Whitney U-test or the probability test based on cycle threshold (T_{stdev}). When specimens had one or two replicate(s) crossed T_{stdev}, the specimens were re-tested in quadruplicate.
2. This table lists tissue specimens that were determined CWD positive using the Mann-Whitney U-test or had at least one replicate crossed T_{stdev}.
3. A total of 8 replicates were tested when specimens were subjected to re-testing.

Table S3: Screening chronic wasting disease in 104 white-tailed deer by RT-QuIC based on MPR[1] and ELISA

RT-QuIC					
	Welch T Test		Probability test		
ID[2]	P-value	Pos/Neg	#Pos/#tested[3]	Pos/Neg	ELISA
Obex-1	0.192	Neg	1/8	Neg	Neg
Obex-7	4E-05	Pos	4/4	Pos	Pos
**Obex-10**	0.029	Pos	0/4	Neg	Neg
**Obex-12**	0.009	Pos	0/4	Neg	Neg
Obex-18	0.195	Neg	1/8	Neg	Neg
Obex-19	0.007	Pos	7/8	Pos	Pos
Obex-20	4E-04	Pos	4/4	Pos	Pos
Obex-25	0.240	Neg	1/8	Neg	Neg
Obex-29	0.141	Neg	2/8	Neg	Neg
Obex-34	0.259	Neg	1/8	Neg	Neg
Obex-37	0.297	Neg	1/8	Neg	Neg
Obex-45	0.008	Pos	4/4	Pos	Pos
Obex-55	0.009	Pos	4/4	Pos	Pos
Obex-56	0.207	Neg	2/8	Neg	Neg
Obex-75	0.466	Neg	1/8	Neg	Neg
Obex-80	0.312	Neg	2/8	Neg	Neg
Obex-97	0.535	Neg	1/8	Neg	Neg
Obex-100	0.614	Neg	1/8	Neg	Neg
RLN-7	0.004	Pos	4/4	Pos	Pos
RLN-8	0.099	Neg	3/8	Neg	Neg
RLN-9	0.316	Neg	2/8	Neg	Neg
RLN-18	0.492	Neg	1/8	Neg	Neg
RLN-19	2E-04	Pos	4/4	Pos	Pos
RLN-20	7E-05	Pos	4/4	Pos	Pos
RLN-21	0.346	Neg	1/8	Neg	Neg
RLN-28	0.753	Neg	1/8	Neg	Neg
RLN-35	0.632	Neg	1/8	Neg	Neg
RLN-38	0.400	Neg	1/8	Neg	Neg
RLN-39	0.760	Neg	1/8	Neg	Neg
RLN-40	0.451	Neg	1/8	Neg	Neg
**RLN-43**	0.005	Pos	0/4	Neg	Neg
RLN-45	2E-04	Pos	4/4	Pos	Pos
**RLN-49**	0.010	Pos	0/4	Neg	Neg
**RLN-54**	0.005	Pos	0/4	Neg	Neg
RLN-55	0.002	Pos	4/4	Pos	Pos
**RLN-57**	0.012	Pos	0/4	Neg	Neg
**RLN-58**	0.002	Pos	0/4	Neg	Neg
**RLN-60**	0.016	Pos	0/4	Neg	Neg

RLN-61	0.104	Neg	2/8	Neg	Neg
RLN-62	0.133	Neg	1/8	Neg	Neg
**RLN-63**	0.031	Pos	0/4	Neg	Neg
RLN-77	0.207	Neg	1/8	Neg	Neg
RLN-84	0.273	Neg	1/8	Neg	Neg
RLN-88	0.326	Neg	1/8	Neg	Neg
RLN-92	0.264	Neg	1/8	Neg	Neg
RLN-100	0.355	Neg	1/8	Neg	Neg

1. Obex and RLN tissue specimens from 104 white-tailed deer were tested by RT-QuIC in quadruplicate with an assay duration of 102 cycles (29 hours) and 110 cycles (32 hours) for obex and RLN, respectively. CWD positivity was determined using the Welch's t-test or the probability test based on MPR threshold (T_{MPR}) of 3.36 and 2.00 for obex and RLN, respectively. When specimens had one or two replicate(s) crossed T_{MPR}, the specimens were re-tested in quadruplicate. False-positive samples by the Welch T test are bolded.
2. This table lists tissue specimens that were determined CWD positive using the Welch's t-test or had at least one replicate crossed T_{MPR}.
3. A total of 8 replicates were tested when specimens were subjected to re-testing.

Table S4: Replicate-level comparison of RT-QuIC screening of 104 white-tailed deer at various assay durations and classifier thresholds

Tissue	Classifier	Assay Duration (h)	Signal threshold[1]	TP[2]	TN[3]	FP[4]	FN[5]	% Agreement[6]
Obex	Ct	33	T_{stdev}	20	369	27	0	93.51%
RLN	Ct	30	T_{stdev}	20	380	16	0	96.15%
Obex	Ct	40	T_{stdev}	20	352	44	0	89.42%
RLN	Ct	40	T_{stdev}	20	361	35	0	91.59%
Obex	MPR	29	$T_{MPR} = 3.36$	19	384	12	1	96.88%
RLN	MPR	31	$T_{MPR} = 2.00$	20	382	14	0	96.63%
Obex	MPR	40	$T_{MPR} = 3.36$	20	356	40	0	90.38%
RLN	MPR	40	$T_{MPR} = 2.54$	20	365	31	0	92.55%

1. T_{stdev} was defined per plate as the first-cycle reading of all tested wells plus 10 sample standard deviations. T_{MPR} was defined per sample as a ratio to the reading in the 4th cycle (~52 min).
2. TP was defined as a replicate that crossed T_{stdev} or T_{MPR} within the assay duration and was derived from an ELISA-positive sample.
3. TN was defined as a replicate that did not cross T_{stdev} or T_{MPR} within the assay duration and was derived from an ELISA-negative sample.
4. FP was defined as a replicate that crossed T_{stdev} or T_{MPR} within the assay duration and was derived from an ELISA-negative sample.
5. FN was defined as a replicate that did not cross T_{stdev} or T_{MPR} within the assay duration and was derived from an ELISA-positive sample.
6. % agreement was calculated as $\frac{TP+TN}{TP+TN+FP+FN}$

Table S5: Contingency tables for a replicate-level McNemar test[1] between optimized assay durations and a 40 h assay duration

Threshold	Tissue	Pos (40 h) & Pos (optimized)	Pos (40 h) & Neg (optimized)	Neg (40 h) & Pos (optimized)	Neg (40 h) & Neg (optimized)	McNemar's Statistic	p^2
T_{stdev}	Obex	31	33	352	0	31.0	2.54E-08
T_{stdev}	RLN	36	19	361	0	17.1	3.64E-05
T_{MPR}	Obex	31	29	356	0	27.0	2.00E-07
T_{MPR}	RLN	40	11	365	0	9.1	2.57E-03

1. Obex and RLN tissue specimens from 104 white-tailed deer were tested by RT-QuIC in quadruplicate with an assay duration of 33 hours for obex and 30 hours for RLN with T_{stdev}, and 29 hours and 32 hours for obex and RLN with T_{MPR}, respectively. T_{stdev} was defined per plate as the average of all tested wells plus 10 standard deviations. T_{MPR} was defined per well as the maximum fluorescence over the fluorescence at the fourth reading, and was set to 3.36 and 2.00 for obex and RLN at the optimized durations, respectively, and 3.36 and 2.54 at 40 hours, for obex and RLN, respectively.
2. The probability value (P) was calculated using 1 degree of freedom.
